# An unusual diagnosis for persistent diarrhoea and vomiting 

**Published:** 2017

**Authors:** James Nott, Asad Khan, Ravi Madhotra, George MacFaul, Kamran Rostami

**Affiliations:** Department of Gastroenterology, Milton Keynes University Hospital, Milton Keynes, UK. 2016

## Abstract

Identifying the etiology of chronic diarrhoea might be challenging in some patients, and before a diagnosis is made these patients may spend a substantial length of time with unresolved symptoms leading to uncertainty and anxiety that is severely impairing their life quality. A 45-year-old female was referred by her general practitioner with a 5-year history of increasingly frequent episodes of cyclical diarrhoea, vomiting, abdominal pain and intermittent palpitations. Contrast CT Abdomen/Pelvis revealed a 36x33x46 mm mass in the aorto caval region of her retro-peritoneum, just above the bifurcation.  On the basis of her symptoms, CT findings and an elevated plasma metanephrine level of 2314pmol/L (normal range 80 – 510pmol/L), it was at this point a likely diagnosis of a phaeochromocytoma was made. The retroperitoneal mass was successfully resected, and the histology confirmed a Phaeochromocytoma. Her symptoms rapidly improved and she made a good recovery. This unusual case highlights some of the dilemmas that arise when investigating patients with chronic and recurrent diarrhoea and vomiting.

## Introduction

Pheochromocytomas are rare and often sporadic tumours of catecholamine-secreting chromaffin cells, usually present within the adrenal medulla, but also may occur elsewhere (1). Early diagnosis of pheochromocytoma is vital, because of developing crisis of hypertension or shock and eventually death. Eight to ten percent of tumours are malignant, and the diagnosis need to awareness of clinicians (2). In this article, we describe a case of Pheochromocytoma with unusual presentation. 

## Case Report

A 45-year-old female was referred by her general practitioner with a 5-year history of increasingly frequent episodes of cyclical diarrhoea, vomiting, abdominal pain and intermittent palpitations. Her bowels would open up to ten times a day and she described them as ‘explosive’ and loose in nature.  There was no blood or mucus per rectum, or any nocturnal symptoms. She vomited approximately fifteen times a day and experienced non-specific abdominal pain. These episodes would last for several days and occur on a monthly basis. Other than a Laparoscopic Cholecystectomy, she had no significant medical, surgical or family history, and took no regular medications. 

**Fig.1:**
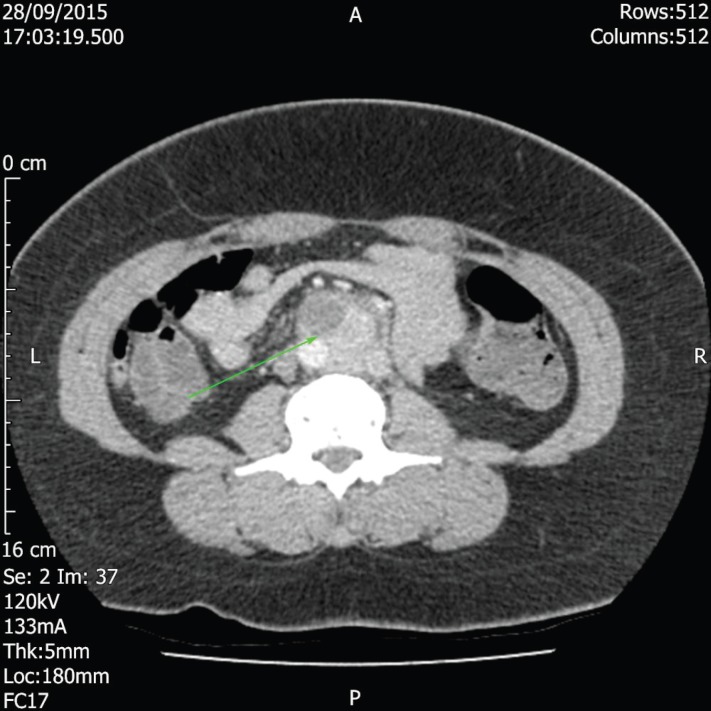
CT Image demonstrating retroperitoneal mass

The patient’s persistent cyclical symptoms resulted in 7 identical short admissions to hospital, during which she was diagnosed with ‘non-specific abdominal pain’. Each time symptoms settled with conservative therapy, and she was discharged with the reassurance of normal routine bloods and simple abdominal imaging.  During her latest admission, she was found to be hypertensive with a systolic of 160mmHg, which spontaneously resolved prior to discharge, and was not thought to be clinically relevant at the time.

Although being actively investigated as an outpatient, the patient requested a second opinion and so a referral was made to the regional tertiary centre. She was thoroughly investigated and returned to Milton Keynes Hospital with a list of possible differential diagnoses including Cyclical Vomiting Syndrome, Bile Salt Malabsorption and Visceral Hypersensitivity. No records of any work up are available. 

Since the patient had a history of laprascopic cholecystectomy, it was hypothesised that her pain might be explained by a retained gallstone. However, two MRCPs were normal with no retained gallstone or common bile duct dilation was found. Abdominal and pelvic ultrasounds were also normal.  Therefore, the pain was considered to be originating from her renal tract and although the first CT KUB was normal with no evidence of renal calculi or hydronephrosis, a subsequent CT KUB confirmed the peri-aortic lymphadenopathy. This incidental finding prompted a Contrast CT Abdomen/Pelvis in September 2015, which found a 36 x 33 x 46mm mass in the aorto caval region of her retro-peritoneum, just above the bifurcation. 

While local and regional MDT meetings were being sought, the patient continued to be investigated for a co-existing pathology. Both OGD and Colonoscopy were normal, in addition to blood tests including thyroid function, autoimmune profile, porphyria, CA19-9 and HIV. She demonstrated no evidence of pancreatic insufficiency with a faecal elastase of >500µg/g and TB Elispot negative. The MDT outcome was to biopsy the retroperitoneal mass, but due to the complexity and proximity of the lesion to the aorta, she was referred to the national sarcoma team in Birmingham. 

 On the basis of her symptoms, CT findings and an elevated plasma metanephrine level of 2314pmol/L (normal range 80 – 510pmol/L), it was at this point a likely diagnosis of a Phaeochromocytoma was made. Furthermore, a meta-iodobenzylguanidine (MIBG) CT was performed and showed that the retroperitoneal aorto caval mass was clearly avid, in keeping with an ectopic Phaeochromocytoma. There was no abnormal activity elsewhere in the abdomen, and particular within the adrenal glands. She was subsequently reviewed by the Endocrinology team who commenced Doxazocin 4mg three times daily in order to control her hypertension, and she was listed for surgery.  Despite a very labile systolic blood pressure, fluctuating between 40-300mmHg, the retroperitoneal mass was successfully resected, and the histology confirmed a Phaeochromocytoma (Para-ganglioma with lymph node metastases (3/9), Ki-67 less than 3%, less than 1/12 HPF mitosis). Her symptoms rapidly improved and she made a good recovery.

## Discussion

This unusual case highlights some of the dilemmas that arise when investigating patients with chronic and recurrent diarrhoea and vomiting. It raises the question as to whether we should be routinely screened for Phaeochromocytomas in these cases.

Pheochromocytomas are rare and often sporadic tumours of catecholamine-secreting chromaffin cells, usually present within the adrenal medulla, but also may occur elsewhere. Through over stimulation of α-/β-adrenoceptors, they classically present with a symptom triad of paroxysmal headaches, tachycardia and sweating^1^. Nausea, anxiety and diarrhoea may also be experienced. Diagnostic tests include 24-hour urinary and plasma metanephrine, and less frequently, urinary vanillymandelic acid (VMA) levels^2^. However, increasing numbers are diagnosed incidentally by CT or MRI imaging^2^. Timely surgical resection with pre-operative optimisation through α/β blockade medication is the treatment of choice, and often results in rapid resolution of symptoms^2^. If advanced, there is a role for chemotherapy. 

Benign pheochromocytomas have an excellent prognosis with a 5-year survival rate greater than 95%, but require lifelong follow-up due to disease recurrence, especially with large tumours (>50mm)^3^. 

Phaeochromocytomas are extremely rare, however as this case demonstrates, they should be included within the differential diagnosis for patients with recurrent episodes of diarrhoea and vomiting.
